# Skeletal Muscle and the Effects of Ammonia Toxicity in Fish, Mammalian, and Avian Species: A Comparative Review Based on Molecular Research

**DOI:** 10.3390/ijms21134641

**Published:** 2020-06-30

**Authors:** Emily Miramontes, Paul Mozdziak, James N. Petitte, Magdalena Kulus, Maria Wieczorkiewicz, Bartosz Kempisty

**Affiliations:** 1 Prestage Department of Poultry Science, North Carolina State University, Raleigh, NC 27695, USA; emiramo@ncsu.edu (E.M.); jnppo@ncsu.edu (J.N.P.); 2 Department of Veterinary Surgery, Institute of Veterinary Medicine, Nicolaus Copernicus University in Torun, 87-100 Torun, Poland; magdalena.kulus@umk.pl (M.K.); bkempisty@ump.edu.pl (B.K.); 3 Department of Basic and Preclinical Sciences, Institute of Veterinary Medicine, Nicolaus Copernicus University in Torun, 87-100 Torun, Poland; maria.wieczorkiewicz@umk.pl; 4 Department of Anatomy, Poznan University of Medical Sciences, 60-781 Poznan, Poland; 5 Department of Histology and Embryology, Poznan University of Medical Sciences, 60-781 Poznan, Poland; 6 Center of Assisted Reproduction, Department of Obstetrics and Gynecology, Masaryk University, 62500 Brno, Czech Republic

**Keywords:** muscle, fish, avian, mammal, ammonia, myostatin

## Abstract

Typically, mammalian and avian models have been used to examine the effects of ammonia on skeletal muscle. Hyperammonemia causes sarcopenia or muscle wasting, in mammals and has been linked to sarcopenia in liver disease patients. Avian models of skeletal muscle have responded positively to hyperammonemia, differing from the mammalian response. Fish skeletal muscle has not been examined as extensively as mammalian and avian muscle. Fish skeletal muscle shares similarities with avian and mammalian muscle but has notable differences in growth, fiber distribution, and response to the environment. The wide array of body sizes and locomotion needs of fish also leads to greater diversity in muscle fiber distribution and growth between different fish species. The response of fish muscle to high levels of ammonia is important for aquaculture and quality food production but has not been extensively studied to date. Understanding the differences between fish, mammalian and avian species’ myogenic response to hyperammonemia could lead to new therapies for muscle wasting due to a greater understanding of the mechanisms behind skeletal muscle regulation and how ammonia effects these mechanisms. This paper provides an overview of fish skeletal muscle and ammonia excretion and toxicity in fish, as well as a comparison to avian and mammalian species.

## 1. Introduction

Elevated levels of ammonia may lead to sarcopenia, or muscle wasting, and this has been linked to increased myostatin expression, a negative regulator of muscle growth. Most of the pre-existing research has focused on mammalian and avian models for examining the mechanism of ammonia toxicity, but ammonia toxicity in fish is not extensively understood. Some fish, particularly air-breathing fish, have a higher ammonia tolerance than mammals [1,2,3]. Fish also utilize a different mechanism of ammonia excretion than mammals and avians. Understanding the underlying physiology of fish ammonia metabolism and the effect of ammonia on fish skeletal muscle and other organ systems could lead to therapeutic approaches to hyperammonemia secondary to liver disease in human medicine. Since fish are an important source of food, understanding the impact of ammonia on skeletal muscle mass and quality is also important for optimizing feed and the environment of fish used for food production.

## 2. Skeletal Muscle Fibers

**Figure 1:**
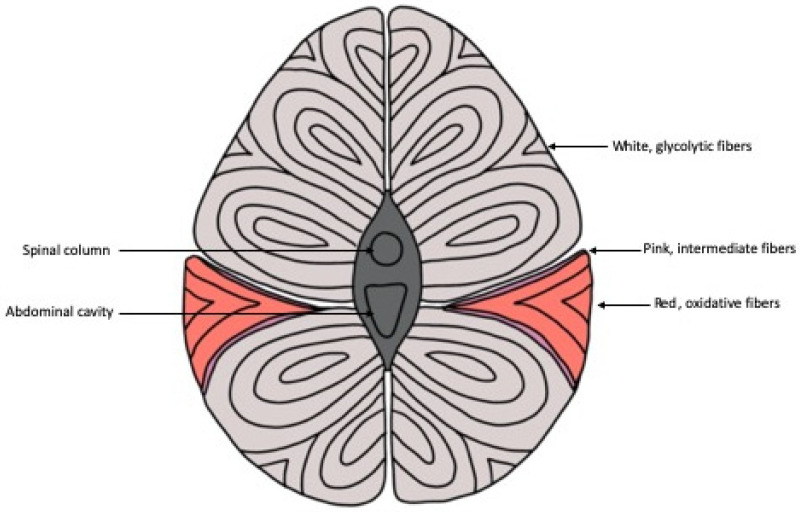
Schematic representation of the cross section of fish musculature showing the distribution of skeletal muscle fibers throughout the body. The thin section of pink muscle fibers in between the white and red muscle fibers is not present in every fish species.

There are three main classifications of muscle fiber types in fish: white, red and pink muscle fibers (Figure 1) [4]. White muscle runs the length of the fish, composes approximately 70% or more of the myotome of most fish and is typically the largest of the fibers, ranging from 50 to 100 µm [5,6,7]. Similar to mammals, white muscle in fish has low levels of myoglobin, vascularization and mitochondria content [8]. This type of muscle is typically recruited during faster swimming states and short-burst swimming sessions utilizing anaerobic metabolism [9,10,11]. Converse to white muscle, red muscle comprises at most 10% of the myotome in a thin section along the lateral line and is categorized as slow-twitch muscle, with high myoglobin content and vascularization [5,8]. In contrast to white muscle, the distribution of red muscle is higher anteriorly, decreases caudally and utilizes aerobic metabolism to sustain longer bouts of swimming, typically at lower speeds than white muscle [9,11,12].

In fish, a third type of muscle fiber called the pink muscle has been identified [13]. It is aptly named, as this muscle, situated in between red and white muscle fibers, has intermediate features in most aspects of its physical appearance and physiological functioning between the abovementioned fibers [13,14]. Pink muscle fibers are typically recruited at intermediate swimming speeds as well as alongside red muscle fibers for sustained swimming at high speeds [14]. The amount of pink fiber type, if it exists in a particular species at all, varies and depends on the swimming needs of that particular species [13]. Pink fibers can be identified histochemically through the fiber’s stability during alkaline preincubation before myofibrillar ATPase staining [15].

Mammals and avians, on the other hand, classify muscle fibers slightly differently. Similar to fish, mammalian muscle fibers are classified as either red or white based on appearance during SDH staining and their metabolic properties [16]. However, while fish have the intermediate pink fibers, some red and white muscle fibers in mammals exhibit metabolic properties of either red or white fibers but have intermediate twitch speeds [17]. On the other hand, in avians, muscle fibers are primarily classified by their metabolic properties, using ATPase staining to identify fast- and slow-twitch muscles and SDH staining to identify their metabolic state [18,19]. Birds and mammals also differ from fish in their distribution of muscle fibers. While fish have more distinct sections of red and white muscle, avian muscle is more heterogenous, and muscle groups have a mixture of all three types of muscle fiber in a single muscle [20].

## 3. Skeletal Muscle Growth

Skeletal muscle accounts for approximately 60% of the total body mass in fish [4]. Alongside its main role of facilitating movement, skeletal muscle also plays a role in metabolism, hormonal control and blood sugar control. Fish muscle also plays an important role in food production and the growth and quality of fish skeletal muscle is of interest to the aquaculture industry [21]. Muscle growth and development in fish species is different from that of mammals, particularly in postnatal hyperplasia (increase in cell number) and hypertrophy (increase in cell size). In fish, both hyperplasia and hypertrophy persist throughout postnatal growth into adulthood [22], while mammals exhibit hyperplasia in embryonic skeletal muscle growth and hypertrophy in postnatal growth [23,24]. Studies done in rainbow trout and carp revealed the persistence of hyperplasia into adulthood, with slight differences in which type of growth predominates based on body length, fiber type, and the location of the muscle sample studied [25,26,27]. Different phases of hyperplasia have been identified in fish and the timing of when these phases begin and end vary between species [28]. In early development, during the larval stage, hyperplasia dominates growth and occurs in the proliferative zones found in embryonic muscle growth [13]. Later in life, during juvenile and adult growth, hypertrophy tends to dominate [13,29]. However, it has been found that some fish, especially those with larger terminal body sizes, have a secondary wave of hyperplasia that persists into adulthood, giving the muscle a mosaic appearance in transverse sections with an intermingling of small and large fibers in the muscle cross section [13,30,31]. Fish that are relatively small will either have this phase greatly reduced or absent entirely during muscle growth [32]. The interplay between hyperplasia and hypertrophy throughout the lifespan of fish causes indeterminate growth, which causes constant growth in body length and mass, as well as the wide range of terminal body sizes across different species [33,34].

Hypertrophy and hyperplasia in fish have been shown to be due to the recruitment of satellite cells [25]. Satellite cells are quiescent cells that exist in differentiated muscle tissues between the sarcolemma and basal lamina. These cells in adult muscle express paired box 7 or PAX7 and this transcription factor is thought to maintain the pluripotency of the satellite cells existing under the basal lamina of muscle. [35,36]. In mammals, the activation of satellite cells is achieved through a basic helix-loop-helix subfamily of proteins called myogenic regulatory factors [36]. Like embryonic myoblasts, muscle satellite cells express myogenic regulatory factor 5 (Myf5), which commits the cell to a muscle cell fate [37,38]. Myogenic determination factor 1 (MyoD) is expressed in cells shortly after Myf5 and together, these two transcription factors initiate determination into myoblasts [36,39]. Differentiation of the myoblasts into multinucleated myotubes is then initiated and regulated by myogenin and myogenic factor 4 (MRF4) [40,41,42]. The pattern of MRF expression previously described has also been found in fish species, such as zebrafish [43], carp [44], rainbow trout [45] and brown trout [46]. Slight variations in gene expression across species is likely due to the differences in the types of growth each species will utilize, especially since larger fish have shown to have hyperplastic growth persist for a longer period of time, as compared with smaller fish.

**Figure 2:**
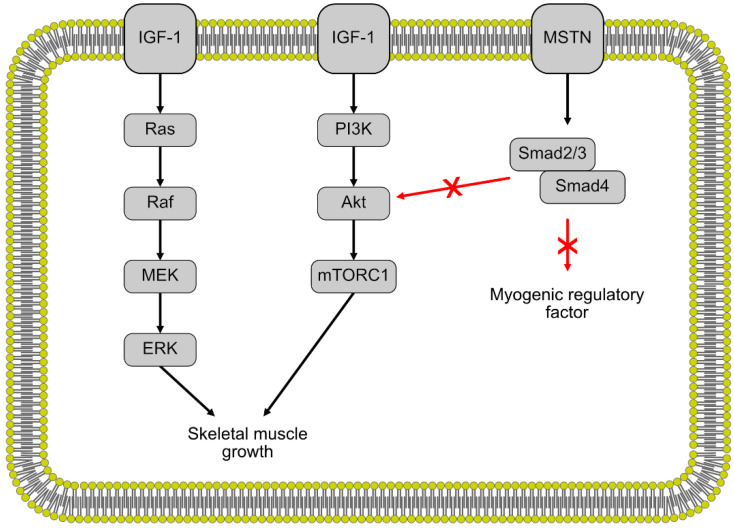
The regulation of skeletal muscle growth by the insulin-like growth factor 1 (IGF-1) and myostatin pathways. Black arrows indicate stimulation white red arrows indicate inhibition.

There are two main regulatory pathways for muscle growth in fish, mammalian and avian species (Figure 2). First, the insulin-like growth factor 1 (IGF-1) pathway is a positive regulator for muscle growth and promotes the proliferation of myocytes [22,47]. IGF-1 regulates muscle growth through the Akt/Protein Kinase B and rapamycin target pathways (mTOR) [48]. IGF-1 also induces muscle cell proliferation through the mitogen-activated kinase and extracellular signal-regulated kinase (MAPK/ERK) pathway [49,50]. Studies done in gilthead seabream and salmon have shown a correlation between higher levels of plasma IGF-1 and increased growth, similar to what has been observed in mammals [51,52]. Mammalian IGF-1 injected into coho salmon had a positive effect on growth, exhibiting a certain level of conservation of the IGF-1 regulation pathway between fish and mammals [53]. Differences in IGF-1 levels and consequently, differences in growth were observed in fast-growing and slow-growing fish, as well as warm-water and cold-water fishes, indicating some environmental and species variation in muscle growth across different fish [51].

## 4. Myostatin

The second regulatory pathway is the myostatin/Smad pathway, a negative regulator of muscle growth. Myostatin is a member of the transforming growth factor (TGF)-β superfamily and contributes to the regulation of muscle growth and development [54]. Myostatin negatively regulates muscle growth through the activation of the Mstn/Smad pathway, inhibiting the transcription of the MRFs that regulate muscle cell differentiation and proliferation [55,56]. Myostatin also negatively regulates muscle growth through inhibiting the Akt/mTOR pathway, thereby slowing protein synthesis [57,58]. In mammals, the double-muscle phenotype, a type of muscular hypertrophy, is the result of myostatin null mutations in mice [54] and cattle [59,60]. This double-muscle phenotype has also been induced in zebrafish [61,62], medaka [18] and trout [63] through the inhibition of myostatin expression. These results indicate a similar function of myostatin during muscle growth and development between fish and mammals. 

Multiple myostatin isoforms have been isolated from different fish species, which is not commonly observed in mammals. In studies in trout [64], Atlantic salmon [65], brook trout [66], zebrafish [67] and gilthead seabream [68], two isoforms of myostatin for each species have been identified. The different myostatin isoforms were also found to be expressed differentially among the species. However, different functions of these isoforms have not been identified in fish. The occurrence of multiple copies of the myostatin gene could be potentially due to the occurrence of polyploidy during the evolution of different fish species [69]. The mature myostatin sequence in fish is well conserved compared with mammals, with about 90% similarity, despite only about 60% similarity in the propeptide [70]. The differences between fish and mammalian prodomains and promoter sequences and other regulatory elements could be responsible for the differential expression of myostatin between fish and mammals [70].

In mice, myostatin expression is high in the muscle [54] and low in the adipose tissues [54], mammary tissues [71] and cardiomyocytes [72]. In fish, myostatin is expressed across multiple tissues including muscle, cardiomyocytes and adipose. In seabream, expression was also found in the brain, eye, intestines and kidneys [68]. Rodgers et al. [73] found that tilapia and white bass expressed myostatin in the muscle, eyes, ovaries, gut, brain and heart. In zebrafish and seabream, myostatin was localized to the brain, skeletal muscle, gills, kidneys, intestines and liver [74]. Since fish express myostatin across multiple tissues, as opposed to the more restrictive expression seen in mammals, it could indicate a wider range of function of the protein in fish.

In mammals, myostatin expression has only been found in fast-twitch muscle fibers [75]. However, studies on different teleosts have found that myostatin is expressed differently across the different muscle fibers [66]. Roberts and Goetz (2001) found that brook trout, king mackerel and yellow perch myostatin expression was localized to red muscle, while little tunny had expression only in white muscle, and mahi mahi had expression in both red and white muscle [66]. Rescan et al. (2001) found that myostatin 1 in trout was expressed in slow- and fast-twitch muscle and myostatin 2 was expressed only in slow-twitch muscle [64]. This difference in myostatin expression between muscle fiber types could be due to the correlation of muscle fiber type ratios and locomotion needs of different fish species [66].

## 5. Ammonia

Ammonia is produced through the catabolism of proteins and specifically through the breakdown of amino acids, typically from diet. Although the gastrointestinal tract is the primary source of amino acids, many different organs and organ systems can produce ammonia [76]. For fish, the pKa of ammonia is approximately 1–2 units above the pH of the blood and intracellular fluid, so about 95% of the ammonia in the body exists as NH_4_^+^ [77]. Since excess amino acids cannot be stored, any excess has to be excreted in a way that is not toxic to the animal. Mammals are a ureotelic species, meaning nitrogenous waste is excreted primarily as urea, while birds are uricotelic and excrete primarily uric acid. Conversely, many fish species are ammoniotelic and excrete nitrogenous waste directly as ammonia.

In mammals, the majority of nitrogen is converted to the nontoxic compound urea through the urea cycle. The urea cycle primarily takes place in liver periportal hepatocytes, in both the mitochondria and the cytosol, using ammonia and bicarbonate to convert ammonia to urea through a series of enzyme-mediated reactions. Alongside the urea cycle, glutamine synthetase (GS) works to form glutamine from ammonia and glutamate, typically in tissues that lack urea cycle enzymes, such as the brain, skeletal muscle, gastrointestinal tract and a small number of hepatocytes [51,78,79]. Glutamate dehydrogenase (GDH) catalyzes a reversible reaction that converts α-ketoglutarate to glutamate, using up free ammonia. GDH also works in the deaminating direction and contributes to the balance of nitrogen levels in the body [80]. Avians, on the other hand, lack urea cycle enzymes and primarily rely on GS to form uric acid [78]. GS in birds is found diffusely in the liver, mimicking the expression pattern of urea cycle enzymes in mammals [81].

Fish display a wider variety of ammonia excretion between species, partly due to the large differences in the environment that different species occupy. While most fish are ammoniotelic, excreting nitrogenous waste as ammonia, a select few fish are ureotelic and, like mammals, excrete nitrogenous waste primarily as urea. For example, the Lake Magadi tilapia in Kenya live in extremely alkaline conditions (pH 10–10.5) and have adapted to contain the full range of urea cycle enzymes in the liver; they will excrete almost all of their nitrogenous waste as urea, since ammonia excretion is impeded in such an alkaline environment [82]. The gulf toadfish is able to switch between ammoniotelic excretion and ureotelic excretion. This fish changes its nitrogen excretion strategy when exposed to air or confined in small spaces, since it spends time on land as well as burrowed under the sand, both of which impede nitrogen excretion via ammonia [83,84]. However, not all air-breathing fish excrete ammonia as urea. The African sharptooth catfish is able to move on to land to avoid drying ponds and can live burrowed underground just as the gulf toadfish. However, these fish lack hepatic urea cycle enzymes and after exposure to high external ammonia concentrations, do not accumulate urea in the blood or ammonia in the body tissues [85].

Despite some exceptions, the large majority of fish excrete nitrogenous waste as ammonia. The gills serve as the primary site of ammonia excretion since it is the primary site of gas exchange and ion transport for fish [86,87,88]. Ammonia (NH_3_) is excreted down the concentration gradient, from the gills, since most aqueous environments have a relatively low concertation of ammonia [89]. This favorable NH_3_ gradient is maintained via acid trapping at the gills, so that NH_3_ is converted to NH_4_^+^ as it leaves the epithelium [90]. Hydrogen ions at the apical membrane are pumped out via H^+^-ATPase and Na^+^/H^+^ exchanger proteins [90,91]. This layer of H^+^ ions at the apical membrane of gill epithelium is also generated by the hydration of CO_2_, either catalyzed or uncatalyzed by carbonic anhydrase [90].

**Figure 3:**
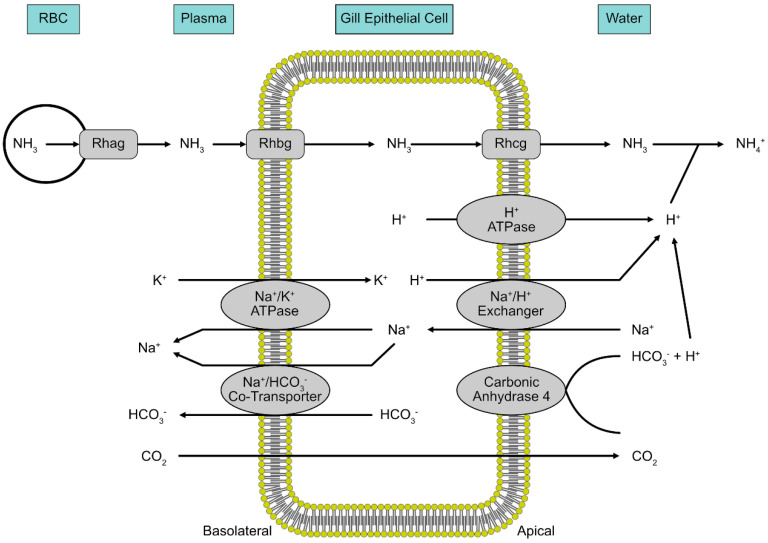
A model of how Rhesus (Rh) glycoproteins are involved with ammonia excretion from the gills of fish. Rhag, Rhbg and Rhcg all participate in the movement of ammonia from the blood into the gill epithelium and out the basolateral side.

More recently, transport proteins have been found in the gills of fish that also participate in the movement of ammonia (Figure 3). Rhesus (Rh) glycoproteins are a family of proteins known to be involved with ammonia transport in various parts of the body tissues in mammals [65,92]. Studies done on pufferfish by Nakada et al. (2007) revealed orthologs to the human Rh glycoprotein family that are localized to the gills of the fish. When these orthologs were used in Xenopus oocytes, ammonia uptake was enhanced as compared with the control [93]. Rh proteins have also been found to be localized to the gills in rainbow trout [94], sea lamprey [86], largemouth bass [95] and zebrafish [96]. Wright and Wood (2009) proposed that the Rhag protein is involved in ammonia transport from erythrocytes to the plasma, followed by Rhbg transport across the basolateral membrane of gill epithelium. The ammonia is then excreted outside the gills by a metabolon on the apical membrane comprised of Rhcg, H^+^-ATPase, Na^+^/H^+^ exchanger and membrane Na^+^ channels [90]. Studies have found that Rh proteins are upregulated during exposure to high levels of ammonia in several species, indicating their involvement in ammonia transport [94,97,98]. Braun et al. (2009) used selective gene knockout of the Rh protein gene in zebrafish and observed a reduction in ammonia transport by about 50%; hence, Rh glycoprotein transporters are necessary for maximal ammonia excretion in the zebrafish [96].

However, similar to mammals and birds, fish also utilize glutamine synthetase (GS) to detoxify ammonia, particularly in the brain [1,99]. Studies in rainbow trout have isolated multiple GS genes and found higher expression of GS in the brain than GDH [100]. Rainbow trout also have increased glutamine concentrations in the brain and liver when exposed to ammonia, which corresponds to increased GS activity [101]. Banerjee et al. (2018) isolated three different isoforms of the glutamine synthetase gene in magur catfish, an air-breathing fish. After exposure to high levels of ammonia, the magur catfish had differential expression of the three copies of the gene, with each localizing to the liver, kidneys, gills, muscle or brain [102]. In the toadfish, it was also found that fish pretreated with methionine sulfoximine (MSO), an inhibitor of GS, were more susceptible to ammonia toxicity than control groups, further supporting the role of GS in detoxifying ammonia [103]. The high level of GS in the fish brain, however, is thought to exert toxic effects during high levels of ammonia exposure, as glutamine can exert negative effects on astrocytes [104]. The exact mechanisms of how fish are able to avoid the neurotoxic effects of glutamine accumulation are still unknown.

## 6. Ammonia Toxicity

In mammals, high levels of ammonia, or hyperammonemia, can be very toxic to various tissues. Most notable is the effect of hyperammonemia on the brain, resulting in a condition called hepatic encephalopathy which is common in patients with liver failure [105]. The increased absorption of ammonia in the brain by the astrocytes leads to increased glutamine production by GS causing osmotic dysregulation, ultimately leading to cellular swelling and metabolic dysfunction [104]. Hyperammonemia also exerts a negative effect on skeletal muscle in mammals, leading to sarcopenia, or muscle wasting [106]. Skeletal muscle, like the brain, contains GS and, during hepatic insufficiency, will take up more ammonia than at basilar levels in an attempt to increase detoxification [107]. Increased ammonia levels have also been connected to an increase in myostatin expression in mammals, which has been shown to be linked with sarcopenia [108,109]. It has been observed that the excess of environmental gaseous ammonia caused by poor ventilation of poultry houses may lead to adverse effects in avians’ eyes or nasal cavities and chronic injuries of the liver [110,111]. However, hyperammonemia in chick embryos has demonstrated potentially beneficial effects on embryonic skeletal muscle [108].

The effects of hyperammonemia on fish are not as extensively studied as is in mammals. However, studies done on fish have shown similar effects of high levels of ammonia as mammals. Many symptoms of hepatic encephalopathy seen in mammals have also been observed in fish, such as hyperexcitability, convulsions and hyperventilation [112,113,114]. Since fish live in aquatic environments, they are also potentially exposed to high levels of environmental ammonia. Fish exposed to high levels of exogenous ammonia also experienced gill hyperplasia [115,116]. That may lead the increased distance that ions must travel across the epithelium to diffuse out of the gills, potentially negatively affecting the efficiency of ammonia excretion in fish [117,118].

However, the exact mechanism of ammonia toxicity is not as well understood in fish as in mammals. Studies on mudskippers [3] found that high levels of ammonia induced high levels of glutamine in the brain but not death, indicating that the mechanism for toxicity in the brain of fish could differ from that of mammals. Similar findings were reported for the African sharptooth catfish [2] and the swamp eel [119]. NH_4_^+^ has also been shown to be able to substitute for K^+^ in K^+^ ion channels in neurons, affecting the membrane potential and excitability of the neuron and could account for the hyperexcitability and convulsions observed in hyperammonemic fish [120,121].

Mammals have shown to respond negatively to high levels of ammonia, resulting in higher levels of myostatin expression and decreased myotube diameter [108]. Conversely, avians exhibited a positive myogenic environment in response to heightened levels of ammonia, with increased myotube diameter and a decrease in myostatin expression [108,122]. Since myostatin appears to have similar functions in both fish and mammals, one hypothesis is that the effects hyperammonemia has on mammalian muscle would mirror those in fish muscle. It has been documented that high levels of ammonia negatively impact growth in fish [123,124]. Dosdat et al. (2003) also found that after sea bass are removed from high-ammonia environments, they exhibit compensatory growth. This indicates that ammonia exerted an inhibitory effect on muscle growth [124]. However, this potential negative impact on the muscle of fish has not been linked to increased myostatin expression, as it has in mammals [108]. The similarities of myostatin functioning between mammals and fish could potentially indicate a role of myostatin in the effect of ammonia toxicity on the growth of fish, but this has not been shown to date.

## 7. Conclusions

Fish, mammals and avians share many similarities in skeletal muscles and ammonia toxicity but also have many differences. It is also well known that fish use a different strategy of ammonia excretion and understanding the ammonia metabolic systems and proteins, such as the Rh proteins, that are involved is crucial for understanding how these fish are able to tolerate high ammonia levels. Some fish have even been found to have higher capacities for ammonia tolerance. Further examination of this tolerance as well as fishes’ ability to effectively mitigate the toxic effects of ammonia could help find new forms of therapy for patients with liver failure. Examining the relationship between ammonia levels in fish and myostatin expression, and the resultant effects on skeletal muscle, will also increase the current understanding of the role of this protein in the effects of hyperammonemia. Further studies on the influence of different levels of ammonia on fish skeletal muscles can also lead to improvement of the quality and quantity of aquaculture industry production.

## Abbreviations

GDH	Glutamate dehydrogenase
GS	Glutamine synthetase
IGF-1	Insulin-like growth factor 1
MRF	Myogenic regulatory factors
MRF4	Myogenic regulatory factor 4
MSO	Methionine sulfoximine
mTOR	Mammalian rapamycin target pathway
Myf5	Myogenic regulatory factor 5
MyoD	Myogenic determination factor 1
MSTN	Myostatin
PAX7	Paired box
Rh	Rhesus
TGF	Transforming growth factor
UOC	Urea-ornithine cycle
